# The roles of protein ubiquitination in tumorigenesis and targeted drug discovery in lung cancer

**DOI:** 10.3389/fendo.2023.1220108

**Published:** 2023-09-19

**Authors:** Zhen Ye, Jingru Yang, Hanming Jiang, Xianquan Zhan

**Affiliations:** ^1^ Medical Science and Technology Innovation Center, Shandong Key Laboratory of Radiation Oncology, Shandong Cancer Hospital and Institute, Shandong First Medical University & Shandong Academy of Medical Sciences, Jinan, Shandong, China; ^2^ School of Clinical and Basic Medicine, Shandong First Medical University & Shandong Academy of Medical Sciences, Jinan, Shandong, China

**Keywords:** lung cancer, ubiquitination, deubiquitination, ubiquitination ligase, ubiquitin-proteasome system, PROTACs, Targeted drug discovery

## Abstract

The malignant lung cancer has a high morbidity rate and very poor 5-year survival rate. About 80% - 90% of protein degradation in human cells is occurred through the ubiquitination enzyme pathway. Ubiquitin ligase (E3) with high specificity plays a crucial role in the ubiquitination process of the target protein, which usually occurs at a lysine residue in a substrate protein. Different ubiquitination forms have different effects on the target proteins. Multiple short chains of ubiquitination residues modify substrate proteins, which are favorable signals for protein degradation. The dynamic balance adapted to physiological needs between ubiquitination and deubiquitination of intracellular proteins is beneficial to the health of the organism. Ubiquitination of proteins has an impact on many biological pathways, and imbalances in these pathways lead to diseases including lung cancer. Ubiquitination of tumor suppressor protein factors or deubiquitination of tumor carcinogen protein factors often lead to the progression of lung cancer. Ubiquitin proteasome system (UPS) is a treasure house for research and development of new cancer drugs for lung cancer, especially targeting proteasome and E3s. The ubiquitination and degradation of oncogene proteins with precise targeting may provide a bright prospect for drug development in lung cancer; Especially proteolytic targeted chimerism (PROTAC)-induced protein degradation technology will offer a new strategy in the discovery and development of new drugs for lung cancer.

## Highlights

1. Ubiquitin-proteasome system is the main pathway of protein degradation in human cells; However, protein ubiquitination is not only involved in protein degradation but also involved in other metabolic regulation. The forms of protein ubiquitination are also diverse.
2. Targeted therapy for ubiquitin systems provides new avenues for developing new drugs for malignant tumors, including lung cancer.
3. Targeted degradation technology serves as a mediator for the connection between ubiquitin ligases and target proteins, which provides new ideas to develop new drugs for malignant tumors, including lung cancer.

## 1 Introduction

### 1.1 The epidemiology of lung cancer

Malignant tumor is a serious problem in public health worldwide and the second main reason of death among the American residents. LC is one of the highest incidence rate tumors worldwide. In the United States, more than 350 people will die of LC every day, which is more than breast cancer, prostate cancer and pancreatic cancer combined, and reached an astonishing 21% of all cancer deaths (1). Worldwide, LC is the ranked first factor for male death and the ranked second factor for female death. The morbidity and mortality rates of LC are highest in high-income countries, such as Europe and North America. Tobacco control measures to prevent and quit smoking can reduce LC deaths (2). Its five-year survival rate is low and varies with clinical stage and regional, ranging from 4% -17% (3). Thus, the incidence of LC is very high, with a poor five-year survival, and the research for better treatment is urgent. Studies have shown that approximately 20% of lung cancer patients experience brain metastasis during diagnosis; throughout the entire course of LC, the probability of brain metastasis is as high as 50%, which increases the mortality rate of patients (4, 5).

### 1.2 Composition and overall function of ubiquitination

Ubiquitin (Ub) is a conservative and widely existed protein with 76 amino acid residues in eukaryotic cells. The Ub-proteasome system (UPS) consists of six key components, including Ub, Ub-activating enzyme (E1), Ub-binding enzyme (E2), UB ligase (E3), deubiquitination enzyme (DUB), and 26S-sized proteasome (6). The number of E1s identified is not very large, Ub-activating enzyme 1 and Ub-like modifier-activating enzyme 6 are in the category of E1s (7). Among the eight E1s identified to date, the enzymes that initiate ubiquitination, SUMOylation, and neddylation are biggest category, and are involved in multiple aspects of cancer biological pathways. To date, more than 40 potential targeted drugs have been reported to target enzymes initiating ubiquitination, SUMOylation, and neddylation, and over 30 clinical trials have been conducted (8).The feature of E2s was a conserved Ub-binding domain that interacts with E1s and E3s (9). Compared to E1s and E2s, E3s have higher specificity in the ubiquitination process (10). Currently, more than 600 species E3s have been identified in the human proteome. DUBs are a large family of proteases. In the human genome, about 100 DUBs were encoded, which were divided into different families per their catalytic domains, the biggest DUB family is cysteine proteases, and the common DUBs are UB C-terminal hydrolases, Ub-specific proteases (USPs), Jab1/Mov34/Mpr1 metalloproteinases, Machado-Joseph disease proteases, ovarian tumor proteases, and a new DUB family of proteases containing MIU (11, 12). What interesting is that E3s and DUBs are potential therapeutic targets for LC therapy due to their high specificity to substrate proteins.

Ubiquitination is an ATP-consumption cascade process that connects the Ub group to the substrate target protein. E1 initially consumes energy ATP, and then binds to Ub group for activation, and then transfers the activated Ub to E2. E3 eventually transfers Ub from E2 to the substrate (13). In a word, Ub conjugation to target protein to degrade this target protein is mediated by an E1 (activation) - E2 (conjugation) - E3 (ligation enzyme) sequential enzyme reaction (14). The completion of ubiquitination process of the target protein will form an isopeptide bond between the Ub C-terminal carboxyl group and the ϵ amino group at lysine residue in the substrate protein, accompanying dehydration and condensation reaction. The above process is a classical ubiquitination process, in addition, there is non-classical ubiquitination, including SidE-mediated ubiquitination independent of E1 and E2, and non-classical ubiquitination sites through the formation of sulfur or hydroxy-bonded esters (Ser/Thr/Cys) (15, 16). Ub ligase is the final and probably the most critical determinant which specific protein undergoes ubiquitination (17). In conclusion, the classical ubiquitination of a substrate protein requires energy consumption, and the ubiquitination of specific proteins is mainly determined by E3s.

In the process of protein maturation after translation of coding genes, most proteins require chemical modifications, including phosphorylation, acetylation, threacylation, glycosylation, and ubiquitination (18). In cells, UPS participates in the degradation of more than 80% proteins (19). Most proteins in human cells undergo ubiquitination at some points in their lifetime (20). Thus ubiquitination is one of the most important protein modifications in cells. It plays multiple effects in cellular pathways, affecting the fate of proteins, signaling and response to various stresses, apoptosis, and necrosis (21, 22). Meanwhile, ubiquitination is essential for a lot of physiological processes, not limited to cell survival, differentiation, and innate and adaptive immunity. Ubiquitination is involved in the functional regulation of immune cells by recognizing receptor signals, mediating innate immune responses, and initiating dendritic cell maturation required for adaptive immune responses. Ubiquitination also regulates the activation, development, and differentiation of T-cells, to maintain effective adaptive immune responses to pathogens and immune tolerance to autologous tissues (23). CDK5 knockdown can mediate Ub degradation of target protein PD-L1 through TRIM21, which thereby improves peripheral immune response to lung adenocarcinoma (LUAD) (24). Therefore, Ubiquitination is also involved in the immune response of tumors. Because of the diverse functions of substrate proteins, ubiquitination of substrate proteins will degrade target proteins, which will produce many biological effects and participate in many biological processes.

UB molecules can also undergo other posttranslational modification of coding genes, such as acetylation and phosphorylation, which indicate the diversity of UB signaling regulation (25). Similarly, the ubiquitination of core components involved in cellular signaling activities controls the conversion of biological signals and cellular outcomes. Similar to kinases, abnormalities in the components of the ubiquitination system lead to a variety of disorders, and can cause illness in severe cases, such as cancer and neurodegenerative diseases (26). The imbalance between ubiquitination and deubiquitination can also cause the occurrence of various diseases, including cancer (27). Overbalanced ubiquitination and deubiquitination participating cancer metabolism in the regulation of signal pathways, transcription factors and so on (27).

The chemical modification of substrate protein is organically linked and can interact with each other. Serine (Ser) and threonine (Thr) contain hydroxyl groups, which are active and easy to be modified by other chemical groups, such as phosphorylation. Phosphorylation at residues Ser and Thr can also mediate the degradation of substrate proteins through UPS pathway (28). For example, SPHK2-mediated phosphorylation of KLF2 promotes the ubiquitination of KLF2 (28). Therefore, if the human body is to be healthy, ubiquitination Ub and deubiquitination need to maintain a dynamic balance, otherwise it will lead to disease.

### 1.3 The modified form of ubiquitination

Ubiquitin can be condensed and linked to the target protein lysine residue through the carboxyl terminal. Ubiquitin molecule contains 7 lysine residues (K6, K11, K27, K29, K33, K48, and K63). Ub binds to the substrate protein in different forms to result in different effects: monoubiquitination may promote the recognition of substrate proteins, complex formation, or allosteric regulation, while K48-linked polyubiquitin is the signal of proteasome degradation. Ubiquitination of K63 connections has non-degrading effects in cell signaling, intracellular transport, DNA damage response, and other environments (20, 29, 30).

The ways that ubiquitination modified proteins are diverse, ranging from monoubiquitination, multiubiquitin chains, and mixed chains (31). Polyubiquitin chains formed by K48 or K11 with substrate proteins usually as a signal of proteasome degradation (32). The ubiquitinated protein can be degraded by proteasome. However, Ubiquitination of the substrate protein does not mean a certain degradation of the protein. The connections between Ub and the substrate protein at different locations play different roles. Compared with a single four-ubiquitin chain, two double-ubiquitin modifications are better signals of degradation (33). In addition to single Ub moiety (mono-ubiquitination), the polyUb chain can be connected by the seven Lysine residues on the ubiquitin chain or N-terminal methionine residues. Thus, eight Ub chains with different structures and functions are formed (20, 29). Multiple short chains (such as double ubiquitins) or branched ubiquitins are better signals for protein degradation than single K48-linked tetraubiquitin (20). The main and most abundant types of protein ubiquitination are monoubiquitination as well as K48- and K63-linked polyubiquitination (34). However, it is difficult to determine the type and number of Ub chains on specific protein substrates in cells (35). Ubiquitination in a protein can be reversed by the catalytic action of a DUB (36). The linkage of different amino acid residues to the substrate protein of Ub can produce different biological effects. It can be seen that ubiquitination of substrate proteins is often linked by lysine residues, and multiubiquitination of multiple short chains is a better signal of protein degradation.

## 2 Ubiquitin-proteasome system plays important roles in pathophysiology and discovery of therapeutic targets of lung carcinoma

Cancer cells have higher protein turnover compared to normal cells. In this context, the ubiquitin-proteasome system (UPS), which degrades most of the cellular proteins in cells, is critical to cancer cells, which is an important drug target for the development of novel cancer therapies (6). UPS is a valuable treasure house for the development of specific drug targets for cancer (32).

### 2.1 E3 enzyme profiling in LC

**Figure 1:**
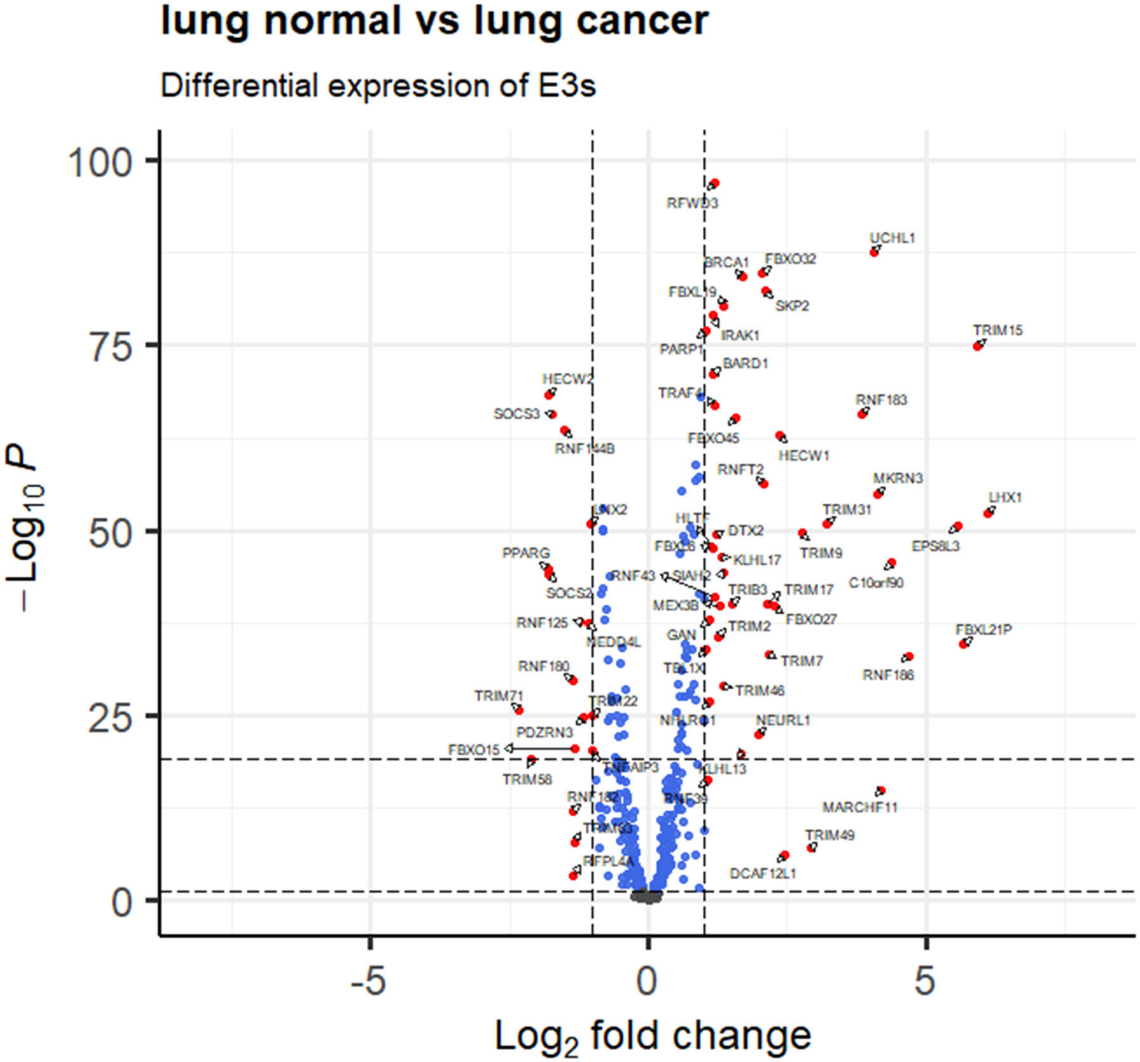
E3 Ub-ligase profiling in lung cancers relative to controls. Differentially expressed E3 genes are determined with transcriptomics data between lung cancers (n =1027) and controls (n=108) from the TCGA database (fold change greater than 1 or less than -1, and p < 0.05).

**Figure 2:**
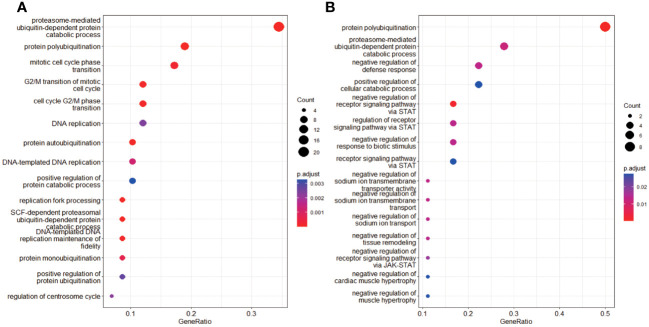
Functional characteristics of differentially expressed E3 Ub-ligase genes in LC. **(A)** GO enrichment analysis of up-regulated E3 genes in LC group. **(B)** GO enrichment analysis of down-regulated E3 genes in LC group.

**Figure 3:**
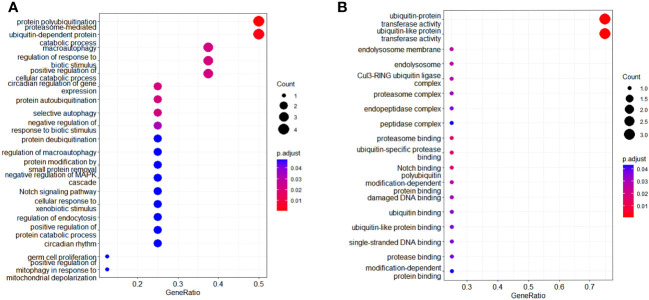
Functional characteristics of differentially expressed E3 Ub-ligase proteins in LC. **(A)** GO enrichment analysis of up-regulated E3 proteins in LC group. **(B)** GO enrichment analysis of down-regulated E3 proteins in LC group.

A total of 414 human E3s in LC were obtained from the UbiBrowser 2.0 database, and were analyzed with the software package DESeq2 (https://www.bioconductor.org/packages/release/bioc/manuals/DESeq2/man/DESeq2.pdf). A total of 77 differentially expressed E3 genes were identified in lung cancers from TCGA database, including 58 up-regulatyed E3 genes and 19 down-regulated E3 genes (**Figure 1**, **Supplemental Table 1**). The up-regulated E3 genes are mainly involved in protein polyubiquitination, proteasome-mediated ubiquitin-dependent protein catabolic process, and mitotic cell-cycle phase transition (**Figure 2A**). The down-regulated E3 genes are mainly involved in protein polyubiquitination, negative regulation of defense response, proteasome-mediated ubiquitin-dependent protein catabolic process, and positive regulation of cellular catabolic process (**Figure 2B**, **Supplemental Table 2**). The E3s protein of differential lung squamous cell carcinoma comes from previous research by our research group (37), with 8 upregulated proteins and 4 downregulated proteins. The up-regulated E3 proteins are mainly involved in, protein polyubiquitination, proteasome-mediated ubiquitin-dependent protein catabolic process (**Figure 3A**). The down-regulated E3 proteins are mainly involved in ubiquitin-protein transferase activity, ubiquitin-like protein transferase activity (**Figure 3B**).

### 2.2 Ubiquitination and the progression of LC

Ubiquitination and deubiquitination processes widely exist in human protein reactions. and if the reaction is not balanced, it can cause many diseases, including LC (38). In LC, abnormalities in the ubiquitination metabolic pathway can lead to many pathological outcomes, including tumorigenesis and progress (39).

Ubiquitination of some proteins may promote the progression of LC (**Table 1**). E3 Ub-ligase called HRD1 could promote lung tumorigenesis by ubiquitination of Sirtuin 2 protein (40). SMURF2 is a HECT family member of E3 Ub- ligases, which can inhibit TGF-b signal pathway in LC cells through ubiquitination-mediated degradation of TGF-b receptor I. MiR-195 and miR-497 can inhibit LC by inhibiting the expression of SMURF2 to inhibit Ub-mediated TGF-b receptor degradation, which suggests miR-497 and miR-1950 as the potential therapeutic targets of lung cancer (41). TRIM59 can induce ubiquitination of ABHD5, leading to its degradation through the ubiquitin protein pathway, and further promote the progression of LC (42). Overexpression of KLHL38 promotes ubiquitination of PTEN, which activates Akt signaling and contributes to the progression of NSCLC (43). TCRP1 promotes the ubiquitination of FOXO3a in cytoplasm, promotes AKT phosphorylation, and blocks FOXO3 nuclear localization, which thereby regulates cell cycle and promotes NSCLC progress (44). TRIM2 is overexpressed in lung adenocarcinoma, which can regulate the degradation of Snail1 through ubiquitin protein pathway to promote the progression of lung adenocarcinoma (53). ACY1 increases PTEN ubiquitination and activates PI3K/AKT signal to promote LC progression (46). In a word, ubiquitination of tumor suppressor gene protein can promote the degradation of the latter to further promote the progress of LC.

**Table 1:** Ubiquitination of substrate proteins and progression of lung cancer cells.

Starting factor	Effect on ubiquitination	Ubiquitination substrate	Effect on lung cancer progression	Reference
HRD1	Increases	Sirtuin 2 protein	Promote lung tumorigenesis	(40)
SMURF2	Increases	TGF-b receptor I	Promote lung tumorigenesis	(41)
TRIM59	Increases	ABHD5	Promote lung cancer progression	(42)
KLHL38	Increases	PTEN	Promote NSCLC progress	(43)
TCRP1	Increases	FOXO3a	Promote NSCLC progress	(44)
TRIM2	Increases	Snail1	Promote NSCLC progress	(45)
ACY1	Increases	PTEN	Promote NSCLC progress	(46)
Knockdown of NEDD4L	Inhibit	CPNE1	Promote lung cancer progression	(47)
PSMD1	Inhibit	PINK1	Promote lung cancer progression	(48)
linc00665	Inhibit	YB-1	Induces tumor-associated angiogenesis	(49)
AFAP1-AS1	Inhibit	c-Myc	Promote lung cancer progression	(50)
TRIM15	Induce	Keap1	Promotes the progression of NSCLC	(51)
HECW1	Induce	Smad4	Promote NSCLC progress	(52)

For some carcinogenic proteins, their ubiquitination is inhibited to weaken their degradation, wich will enhance the progression of LC. Knockdown of Ub ligase NEDD4L can promote the proliferation and transfer of NSCLC cells *in vitro* and *in vivo* (47). PSMD1 inhibits the ubiquitination of PINK1 protein and enhances its stability, promotes the viability of lung adenocarcinoma cells, and inhibits their apoptosis (48). Linc00665 directly interacts with YB-1 protein to inhibit its ubiquitination, activates the YB-1-angpt4-ANGPTL3-VEGFA axis, and induces tumor-associated angiogenesis in lung adenocarcinoma (49). AFAP1-AS1 could inhibit c-Myc ubiquitination and degradation through interaction with Smad nuclear interaction protein 1 (SNIP1), thereby promoting LC cell migration and invasion (50).

Because the ubiquitin-protein system is associated with the occurrence and development of LC, the associated proteins are potential anticancer targets. TRIM15 promotes the progression of NSCLC by maintaining the stability of Nrf2 mediated by Keap1 ubiquitination and degradation, and may be a candidate therapeutic target for NSCLC (51). HECW1 can induce ubiquitination and degradation of Smad4 to promote proliferation, migration, and invasion of NSCLC cells. HECW1 might be a new target for NSCLC treatment (52). UBE2O, an E2/E3 hybrid ubiquitin-protein ligase, promotes LC genesis and radiation resistance by promoting ubiquitination and degradation of Mxi1, which suggests that UBE2O is a potential radiosensitizing target for the treatment of LC (54). RNF8 is an E3 ligase that regulates Akt activation through k63-binding ubiquitination, thereby leading to LC cell proliferation and chemotherapy resistance. RNF8 might be a promising target in precision medicine for LC (55). LncRNA OXCT1-AS1 can block NARF-mediated ubiquitination to stabilize LEF1 and thereby promote the ability of NSCLC to metastasize, which suggests that lncRNA OXCT1-AS1 is a new therapeutic target for NSCLC (56).

### 2.3 Ubiquitination and the inhibition of LC

Ubiquitination of some proteins can inhibit the progression of LC (**Table 2**). NEDD4L is a new E3 ligase of UBE2T that inhibits PI3K-AKT signal transduction by inducing ubiquitination-mediated UBE2T degradation, which could inhibit the progress of lung adenocarcinoma cells (59). The circNDUFB2 inhibits the progression of NSCLC by promoting IGF2BP ubiquitination and down-regulation (57). Loss of TRIM32 can inhibit lung squamous cell carcinoma proliferation, metastasis, and chemotherapy resistance by stabilizing ARID1A (58, 65). E3 ubiquitin-protein MIB2 could inhibit NSCLC by inducing ubiquitination and degradation of Notch1 (60). The overexpression of SIRT3 can inhibit the proliferation of LC cells by increasing the ubiquitination level of mutated p53 (61). The ubiquitination of CIB1 mediated by CHIP can inhibit the metastasis and migration of lung adenocarcinoma (62). Through ubiquitination mechanism, KLHL18 protein inhibits the expression of phosphatidylinositol 3-kinase (PI3K) p85α and PD-L1 protein, and ultimately prevents the immune escape of tumor cells, thus inhibits NSCLC (63). CCDC65 recruits the E3 Ub- ligase FBXW7 that induce ubiquitination degradation of oncogenic transcription factor c-Myc in tumors, thereby inhibiting the proliferation of lung adenocarcinoma (64). It can be seen that oncogene-encoded protein (oncoprotein) is modified by ubiquitination, which leads to the degradation of oncoprotein and thus inhibits the progression of LC. For lung cancer cells A549 and H1975, with RNF115 knockdown, ubiquitinated p53 was significantly reduced, which significantly reduced the proliferation of LC cells and caused cell cycle arrest (65).

**Table 2:** Ubiquitination of substrate proteins and inhibition of lung cancer cells.

Starting factor	Effect on ubiquitination	Ubiquitination substrate	Effect on lung cancer progression	Reference
circNDUFB2	Induce/increases	IGF2BP	Inhibits the progression of NSCLC cancer	(57)
Loss of TRIM32	Inhibit	ARID1A	Inhibit lung cancer progression	(58)
NEDD4L	Induce/increases	UBE2T	Inhibit lung tumorigenesis	(59)
MIB2	Induce/increases	Notch1	Inhibit NSCLC proliferation	(60)
SIRT3	Induce/increases	p53	Inhibit lung cancer proliferation	(61)
CHIP	Induce/increases	CIB1	Inhibit lung adenocarcinoma proliferation	(62)
KLHL18	Induce/increases	p85α/PD-L1	Inhibit lung cancer proliferation	(63)
FBXW7	Induce/increases	c-Myc	Inhibit lung cancer proliferation	(64)
RNF115 knockdown	Inhibit	p53	Inhibit lung cancer proliferation	(65)

### 2.4 Deubiquitination and the progression of LC

Deubiquitination of proteins can promote the progression of LC (**Table 3**). USP5 binds to CCND1, reduces its ubiquitination level and stabilizes the CCND1 protein. This USP5-CCND1 axis could promote NSCLC cell proliferation and colony formation (66). USP7 synergistically activates PI3K-Akt-mTOR pathway to reduce the ubiquitination level of MCL-1 protein and up-regulate MCL-1 protein, and thereby induces lung tumorigenesis (45, 53). Deubiquitination enzyme USP15 and its similar parallel gene USP4 were overexpressed and promoted the proliferation of lung cancer cells by regulating SRSF1 alternative splicing (67).

**Table 3:** Deubiquitination of substrate proteins and the progression of lung cancer.

Starting factor	Effect on ubiquitination	Ubiquitination of substrate proteins	Effect on lung cancer progression	Reference
USP5	Reduce	CCND1	Promote NSCLC progress	(66)
USP7	Reduce	MCL-1	Inducing lung tumorigenesis	(53)
USP15/USP4	Reduce	SRSF1	Promote lung cancer proliferation	(67)
GRP75	Reduce	HMGA1	Promote early LUAD progression	(68)
Deletion of USP11	Induce/increases	ARID1A59	Promote lung cancer proliferation	(58)
USP7	Reduce	SMAD3	Inhibits lung cancer progression	(69)
USP10	Reduce	PTEN	Inhibits NSCLC progression	(70)
USP46	Reduce	PHLPP1	Inhibit lung cancer proliferation	(71)
USP53	Reduce	FKBP51	Inhibits lung cancer progression	(72)

GRP75 directly binds to HMGA1 and inhibits ubiquitination-mediated degradation of HMGA1. Thus, activation of GRP75-HMGA1-JNK-c-JUN signaling pathway is an important mechanism that could promote early lung adenocarcinoma progression (68). USP47 could deubiquitinate BACH1, glyceraldehyde-phosphate dehydrogenase (GAPDH), and hexokinase 2 (HK2), to promote NSCLC progression (73). Deletion of USP11 can promote the development of squamous cell carcinoma by promoting the degradation of ARID1A (65).

Deubiquitination of proteins can inhibit the progression of LC. By catalyzing SMAD3 deubiquitination, USP7 promotes the positive self-regulation of SMAD3, and inhibits p53-deficient lung cancer progression (69). USP10 can inhibit NSCLC proliferation by inhibiting TRIM25-mediated ubiquitination of PTEN (70). USP46 inhibits AKT signaling by antagonizing PHLPP1 ubiquitination, and inhibits LC cell proliferation under normal growing environment and radiation-induced DNA damage. Combination of radiotherapy and AKT inhibitors is a potential therapeutic pathway for USP46 down-regulated LC (71). USP53 is lowly expressed in lung adenocarcinoma, and USP53 deubiquitinates FKBP51, which thereby inhibits tumor growth in lung adenocarcinoma (72).

### 2.5 Ubiquitination enzyme and the treatment of LC

The Ub protease system as the target therapy can improve the sensitivity of LC chemoradiotherapy. Knockout of Ub- ligase WD-repeat and HMG-box DNA-binding protein 1 can inhibit ubiquitination of MAPRE2, thereby which increases the cisplatin sensitivity of LC A549/DDP cells (74). PRMT5 methylates and causes ubiquitination and degradation of Mxi1, which results in radiation resistance (75). CHIP blocks HSP90β interaction with MAST1 to promote the ubiquitination of MAST1, which thereby inhibits the radiation resistance of NSCLC (76). FBW7 is the E3 Ub-ligase of PD-1 protein, which could promote the PD-1 polyubiquitination at K48 of residue Lys233, thus FBW7 accelerated the PD-1 degradation and enhanced *in vivo* anti-tumor immunity (77). E3 ligase FBXW7 promotes ubiquitination of SOX9 and degradation of proteasome to improve the NSCLC cell radiosensitivity (78). Studies demonstrate that RNF115 mediates p53 ubiquitination to predict poor prognosis in patients with lung adenocarcinoma, and RNF115-p53 axis might be an effective therapeutic target of LC (65). In conclusion, some ubiquitination enzymes can be used as targets for the treatment of LC, and the substrates of these enzymes may be tumor suppressor proteins (**Table 4**). 

**Table 4:** Treatment of lung cancer targeting ubiquitinase.

Treatment method or target	The effect of ubiquitination	Effect on lung cancer	Reference
Knockout ubiquitin ligase WD repeat and HMG-box DNA binding protein 1	Inhibits MAPRE2 ubiquitination	increasing the cisplatin sensitivity of lung adenocarcinoma	(74)
PRMT5 methylates	Causes ubiquitization and degradation of Mxi1	Radiation resistance	(75)
CHIP	Promotes the ubiquitination of MAST1	Inhibiting the radiation resistance of NSCLC	(76)
FBW7	Promotes the polyubiquitination of PD-1 protein	Enhances anti-tumor immunity	(77)
FBXW7	Promotes SOX9 ubiquitination	Improves the radiosensitivity of NSCLC cells	(78)
RNF115	Induce p53 ubiquitination	Predicts poor prognosis in lung adenocarcinoma	(65)

### 2.6 Deubiquitination and the treatment of LC

Deubiquitination enzymes, which hydrolyze the isopeptic bonds in Ub and ubiquitin-like protein conjugates, are important regulators of ubiquitin-mediated signaling pathways and candidate drug targets for many diseases, including LC (79). Many deubiquitination enzymes may eventually become therapeutic targets for LC, and some small molecule inhibitors are currently in clinical research stage (38) (**Table 5**). By inhibiting two deubiquitination enzymes, 61 phenocopies duplicate the double knockout of OTUB1/USP8, and play a significant anti-proliferation role in NSCLC cell lines (80). USP37, a member of the deubiquitinase family, can deubiquitinate snail and prevent their degradation, thereby promoting the migration of LC cells. It clearly demonstrates that USP37 might be an effective therapeutic target of LC (81). Knockout of USP51 may reduce cisplatin resistance in LC cells through ubiquitination of ZEB1 (82). FAM188B plays a carcinogenic role by modulating the deubiquitination of FOXM1. Therefore, FAM188B might be an effective therapeutic target to inhibit the progression of LC (83). The highly expressed MiR-135b in NSCLC directly binds to 3’-non-translational region of deubiquitination enzyme CYLD, regulates ubiquitination and activation of NF-κB signaling, which suggests that miR-135b is an effective therapeutic target for NSCLC (84). Deubiquitination enzyme OTUD1 acts as a cancer inhibitor and inhibits erlotinib resistance of NSCLC through YAP1-SOX9-SPP1 axis (85). The first DUB inhibitor VLX1570 is a dual inhibitor for USP14 and UCHL5, which started its clinical drug trials in 2015 (86). VLX1570 can induce G2/M cell cycle arrest and inhibit LC cell proliferation by down-regulating CDK1 and CyclinB1 (87). In conclusion, for deubiquitination enzymes, some of them are LC promoters, and some of them are LC inhibitors, which provides ideas for targeted therapy of LC. 

**Table 5:** Deubiquitination and the treatment of lung cancer.

Treatment method or target	The effect of ubiquitination	Effect on lung cancer	Reference
The double knockout of OTUB1/USP8		Anti proliferation role in NSCLC	(80)
USP37	Deubiquitination of snail	Promoting the migration of lung cancer cells	(81)
Knockout of USP51	Ubiquitination of ZEB1	Reduce cisplatin resistance in lung cancer cells	(82)
FAM188B	Deubiquitination of FOXM1	Plays a carcinogenic role	(83)
miR-135b directly targets CYLD	Regulating ubiquitination and activation of NF-κB Signaling	Promote lung cancer progress	(84)
OTUD1	Deubiquitination of YAP1/SOX9/SPP1 axis	Inhibit erlotinib resistance of NSCLC	(85)
VLX1570	Induce/increases CDK1 and CyclinB1 ubiquitination	Inhibit lung cancer cell proliferation	(86)

## 3 Ubiquitin-proteasome system-based small molecule inhibitors/drugs in lung carcinoma

UPS plays important roles in lung cancer pathophysiology. Strategies for developing antitumor drugs through the UPS include inhibiting ubiquitin-conjugated enzymes and ligases, preventing ubiquitin-binding domains binding to Ub, and designing new proteasome inhibitors (88).

### 3.1 Small molecule compounds participate in ubiquitination to treat LC

The targeted ubiquitination cascade has emerged as a candidate strategy for the treatment of malignant cancer (89). There are some small molecules that affect the ubiquitination pathway and interfere with the treatment of LC (**Table 6**). The combination of immunotherapy and small molecule inhibitors targeting ubiquitination pathway is another promising way for cancer treatment (90).

**Table 6:** Small molecules affect ubiquitination in the treatment of lung cancer.

Small molecules or small molecules participate	Effect on ubiquitination	Substrate protein	Effect on lung cancer progression	Reference
Metformin	Induce/increases	Nrf2	NSCLC to overcome the resistance to chemotherapy	(91)
CC-885	Induce/increases	PLK1	Enhancing the sensitivity of NSCLC to volasertib	(92)
GSK3α- and ARIH1 activators as well as EGFR inhibitors	Induce/increases	PD-L1	Stimulate anti-tumor immunity	(57)
circNDUFB2	Induce/increases	IGF2BPs	Inhibits growth and metastasis of NSCLC cells	(93)
A chimeric anti-ENO1 mAb	Induce/increases	SLUG	Attenuate cancer cell invasion	(94)
BC-DXI-843	Induce/increases	DX2	Induces apoptosis of lung cancer cells	(95)
lncRNA AL355338	Inhibit	ENO1	Promote NSCLC progress	(97)
LINC00476	Induce/increases	SETDB1	Plays a antitumor role in NSCLC	(56)

Some small-molecule substances can increase sensitivity of LC treatment to relieve the disease. Metformin can weaken the ROS defense system in NSCLC through the degradation of Nrf2 mediated by ubiquitination, which is beneficial for NSCLC to overcome the resistance to chemotherapy (91). CC-885 could selectively promote the ubiquitination and degradation of PLK1, thereby making NSCLC more sensitive to volasertib (92). GSK3α- and ARIH1 activators as well as EGFR inhibitors enhance anti-tumor immune activity by triggering ubiquitination degradation pathway of PD-L1, which thereby enhances cancer therapy (54). The circNDUFB2 could increase the ubiquitination level of target protein IGF2BPs, but this effect was suppressed by its mutation. Up-regulated circNDUFB2 can reduce the stability of IGF2BP protein by enhancing degradation mediated by the ubiquitination pathway, which thereby inhibits the metastasis of NSCLC (93).Some small molecules can control LC progression through ubiquitination. A chimeric anti-ENO1 mAb inhibits ENO1-mediated GSK3b inactivation, promotes ubiquitination and degradation of SLUG protein, and thus attenuates cancer cell invasion. This demonstrates the candidate novel antibodies targeting ENO1 for LC treatment (94). BC-DXI-843 degrades DX2 through SiAH1-mediated ubiquitination, and specifically blocks the interaction between DX2 and HSP70, and thus effectively induces apoptosis of LC cells. BC-DXI-843 is a promising small molecule targeted inhibitor for LC (95). LncRNA AL355338 can stabilize ENO1 protein and prevent ubiquitin-mediated degradation, which might be a candidate target for clinical treatment of NSCLC (96). LINC00476 plays a antitumor role in NSCLC by ubiquitination pathway promoting degradation of SETDB1 (56). Ipomoea batatas polysaccharides specifically promotes ubiquitination degradation pathway of PAK1 and blocks its downstream Akt1/mTOR signaling pathway, which thereby inhibits LC proliferation (97). The N,N-bis (5-ethyl-2-hydroxybenzyl) methylamine (EMD) can induce the degradation of c-Myc protein through the ubiquitin-proteasome mechanism. EMD is a potential new approach for the treatment of C-MYC-driven LC (98).Some small molecules can increase the resistance to treatment of LC. Elevated levels of LCETRL3 or LCETRL4 in NSCLC cells lead to inhibition of ubiquitination of TDP43 or EIF2S1, which thereby enhances the stability of proteins TDP43 or EIF2S1, and leads to resistance to tyrosine kinase inhibitors of epidermal growth factor receptor (EGFR) (99). The c-FLIP stabilizes FoxM1 by inhibiting FoxM1 ubiquitination, thereby upregulating FoxM1 expression at the post-transcriptional level, which promotes NSCLC resistance to sulfurstrepton and osimertinib (100). PPDPF interacts with anti-apoptotic protein BABAM2 to block its ubiquitination, which thereby stabilizes BABAM2 and promotes radiation resistance in LC cells (101). When ubiquitination is in an pathological state, the activated biological process will not only promote cancer progression, but also induce neoplastic immune escape (102). BMI1 could promote ubiquitination and degradation of NLRC5, and inhibits HLA class I, which might contribute to the immune escape of NSCLC (103). PAQR4 is overexpressed in LC. If PAQR4 is absent, Nrf2 protein degradation can be promoted through ubiquitination pathway, thereby improving the sensitivity of cancer cells *in vitro* chemotherapy (104). Knockdown of genes involved in cell cycle or protein ubiquitination was able to convert erlotini-resistant LC cells to become sensitive to erlotinib (105). 

### 3.2 E1 enzyme inhibitors

E1 is the least specific component in the Ub system (106). E1 enzyme activates Ub molecules by consuming ATP, which significantly contributes to occurrence and development process of cancer. PYR-41 and PYZD-4409 have been already reported as inhibitors of E1s. PYR-41 reduces Ub protein accumulation, alleviates endoplasmic reticulum stress, inhibits G2/M cell-cycle pregression, and induced cell authophagy and apoptosis (107). Besides inhibiting ubiquitination, PYR-41 can increase total SUMOylation in cells (108). In a leukemia mouse model, intrabitoneal injection of PYZD-4409 reduced tumor growth without adverse toxicity compared to controls. PYZD-4409 induces the death of cancer cells and preferentially inhibits the clonal growth of myeloid leukemia cells (109). MLN4924 was a NeDD8-activating enzyme (**E1**) inhibitor, which was found to inactivate CRL and accumulate CRL substrates to induce the inhibition of cancer cell growth. MLN4924 could effectively inhibit cancer cell growth by inducing apoptosis, senescence, and autophagy, and enhance the sensibility to chemoradiotherapy in a cell environment dependent manner. However, the signaling molecules that determine the fate of cells after MLN4924 treatment is difficult to determine. Cancer cells gradually develop drug resistance to MLN4924 by selecting target mutations (6). Compared to MLN4924, SOMCL-19-133 significantly inhibits Cullin ubiquitination at lower effective concentrations, subsequently causes DNA strands damage and Chk1/Chk2 activation, which leads to cell cycle arrest and apoptosis in cells (87). In addition, SOMCL-19-133 had exhibited strong anti-proliferative activity against human tumor cell lines, approximately 5.31 times stronger than MLN4924. Oral SOMCL-19-133 treatment and subcutaneous injection resulted in significant tumor suppression in a mouse xenograft model. SOMCL-19-133 is a promising preclinical candidate for cancer treatment (87). The E1 enzyme inhibitor TAK-243 could result in the depletion of Ub conjugates, the interruption of signal events, the induction of protein toxicity stress, and in turn lead to the death of cancer cells (110), which showed certain anti-tumor activity in the preliminary study of human xenograft (110). Although E1 enzyme has certain potential for targeted anticancer therapy, due to its poor pharmacokinetic characteristics, no inhibitor has clinical efficacy at present (111). The first neddylation inhibitor, Perfondinistat, has not yet been approved for clinical treatment of tumor. The clinical approval of Pervonilast will encourage study and explore in depth of other E1 inhibitors, particularly those targeting SUMOylation and ubiquitination (8). The Ub-like modifier activator enzyme E1 inhibitor TAK-981, in mice, reduced Ub-like modifier in pancreatic cancer cells in the nanomole range, resulting in G2/M cell cycle arrest and chromosome separation defects. TAK-981 could effectively limit the tumor burden without systemic toxicity in a KPC3 monogene mouse model. *In vivo*, treatment with TAK-981 could increase the proportion of activated CD8 T-cells and natural killer cells in the tumor and peripheral blood, but briefly reduced the number of B cells. The interferon response with TAK-981 treatment in T, B and NK cells was activated. *In vitro* treatment of CD8 T-cells with TAK-981 induces the activation of STAT1 and interferon target genes. This suggests that the targeting TAK-981 in pancreatic cancer is worthy of expectation (112).

### 3.3 E2 enzyme inhibitors

The 185-amino acid length UBE2F protein is a unique E2 UB-coupling enzyme. The use of small molecule inhibitors or genes to target UBE2F-SAG-CRL5 axis will inactivate CRL5, which could induce cell apoptosis. One study demonstrates that UBE2F-SAG-CRL5 axis is a promising effective drug target. Targeting UBE2F may be the preferred method of anticancer treatment (113). CC0651 is the E2 Ub-binding enzyme Cdc34A, which works through capturing the weak interaction between Ub and the E2 donor Ub-binding site. The inhibitor binds to a complex-binding pocket that is formed by Cdc34A and Ub. CC0651 also inhibited the rate of spontaneous hydrolysis of Cdc34A Ub thioester (114). UBV is an effective inhibitor of E2 Ub-conjugating enzyme Ube2k and prevents Ub charging by E2 enzymes and Ub transfer catalyzed by E3. The binding sites of UBVs indicate that they can directly conflict with Ub-activating enzymes and may disrupt their interaction with E3 ligases through allosteric effects (9). An inhibitor of E2 Ub-conjugating enzyme UbcH5c, called DHPO, binds directly to the UbcH5c protein. DHPO can inhibit the pancreatic cancer cell growth, and also inhibit UbcH5c-mediated degradation of I kappa Bα and activation of NF-kappa B, which is essential for its anticancer activity (115). In summary, E2 inhibitors interfere with the interaction between E1 and E2 or between E2 and E3 to affect ubiquitination of substrate proteins. However, targeting E2 still lacks sufficient specificity compared to E3 inhibitors (90).

### 3.4 E3 enzyme inhibitors

Compared to other elements in the Ub protein system, the Ub ligase system has more proteins and has high potential for drug target recognition and validation (116). E3 ligase-mediated degradation of target proteins is highly substrate specific, thus targeting E3 ligases or their substrates is a promising option to develop new drugs. The MDM2 protein is an E3 Ub ligase that could target the p53 protein for proteasomal degradation. The use of Nutlin-3A, an inhibitor of MDM2 to treat MAPK inhibitor-resistant melanoma cells results in a dose-dependent manner to restore FBXW7 expression and p63 degradation and increase the sensitivity of these cancer cells to apoptosis, thus Nutlin-3A as a way to eliminate melanoma resistance to MAPK inhibitors is worthy of further study (117). There are also several E3 enzymes, such as IAPs, FBW7, SKP2, etc., whose inhibitors have not been reported for clinical application (6). However, although the specificity of E3 inhibitors is very high, there is still some ways to go to practical application of clinical patients.

### 3.5 Proteasome inhibitors

*Some* proteasome inhibitors may enhance the efficacy of conventional chemotherapy drugs and induce cytotoxicity and apoptosis (118). Among the inhibitors of Ub proteasome components, proteasome inhibitors were the earliest used for clinic, such as bortezomib, carfilzomib, and oprozomib. Ub proteasome can recognize and degrade Ub-labeled proteins. For example, bortezomib is a good option for high-risk myeloma patients and neck squamous cell cancer (119, 120). MiR-466 can induce the sensitivity of non-small cell lung cancer to bortezomib therapy (99). The high expression of Smurf2 could predict the poor prognosis of lung cancer, while the treatment of bortezomib can significantly down-regulate Smurf2 in lung cancer cells (100).

### 3.6 Deubiquitinating enzyme inhibitors

Ub-specific proteases (USPs) play crucial roles in many types of cancers and are involved in lots of biological processes, and many deubiquitination enzymes (DUBs) are involved in tumor progression as carcinogens. Thereby, targeting specific USPs may be an effective anticancer treatment strategy, and USP inhibitors may be a potential new drugs to treat cancer. Inhibition of USP7 could degradate oncogenic E3 Ub-ligase MDM2, and reactivate tumor suppressor p53. There are two compounds (FT827 and FT671)that can inhibit USP7 with high specificity and affinity, and FT671 can destabilize USP7 substrate, increase p53 level, cause p53 target gene transcription, induce tumor suppressor p21 expression, and inhibit the mice tumor growth (121).

**Figure 4:**
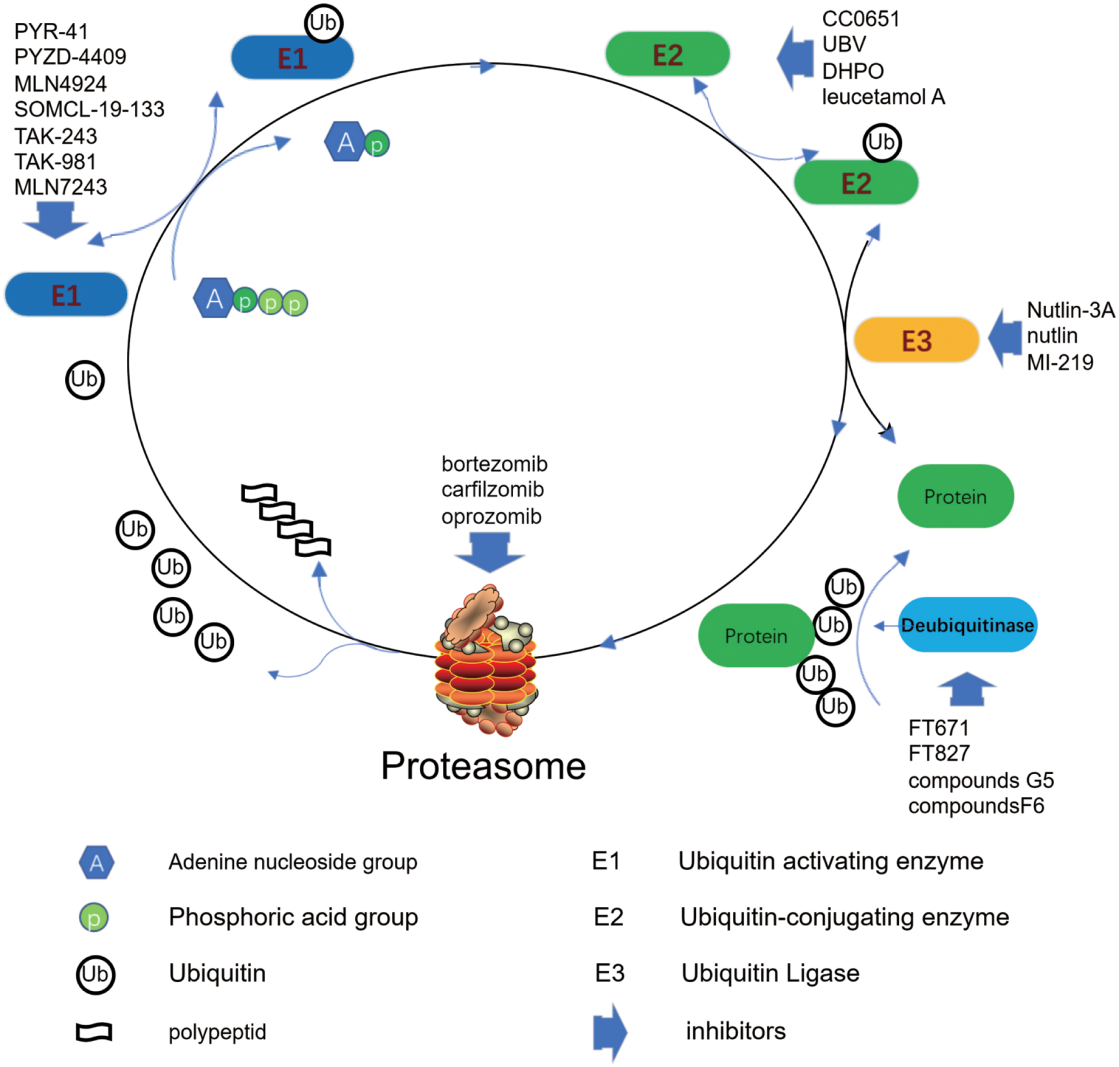
Inhibitors of the ubiquitin proteasome pathway regulate and control protein catabolism.

A lots of targeted drugs based on proteasome, E1, E2, E3, and DUB have been developed for anticancer (**Figure 4**). Inhibitors targeting proteasomes have achieved great success, such as bortezomib, kafizolomi, olazolomi, and isazolomi, with significant clinical efficacy. What is expected is that E1 enzyme inhibitors MLN7243 and MLN4924, E2 enzyme inhibitors leucetamol A and CC0651, E3 enzyme inhibitors nutlin and MI-219, and DUB inhibitors G5 and F6 also offer potential promise (122).

## 4 Targeted protein degradation offers a new strategy in the discovery and development of new drugs for lung cancer

**Figure 5:**
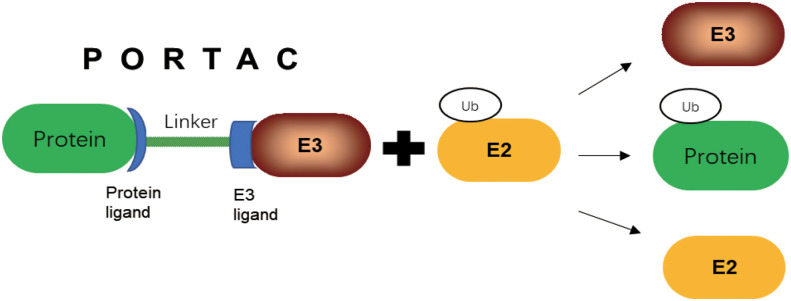
PROTAC mediated E3 target protein ubiquitination.

Proteolytic targeted chimerism (PROTAC)-induced protein degradation is achieved through the selection of natural cellular ubiquitination process. PROTAC technology has been widely applied to lots of pathogenic proteins, and has shown more advantages compared with conventional inhibition strategies. PROTAC is an emerging therapeutic approach, which has the potential to solve difficult to target protein with traditional small-molecule inhibitors, and expand the use of ubiquitin ligase to make precision medicine a success (123). PROTAC has a ligand linker between target protein and an E3 Ub-ligase that was able to link target proteins to E3s. Direct targeting of TFs could have profound effects on cancer cells (124). PROTAC induces proximity between E3 Ub-ligase and target protein, whose process is similar to the proximity effect of enzymes to promote ubiquitination and degradation of the protein of interest (**Figure 5**). By recruiting Ub-ligase E3 to a target protein, PROTACs hijacks the ligase activity for ubiquitination of the interested protein and degradation by the proteasome (125). In addition to small molecule inhibitors, PROTAC offers more opportunities in future in E3 Ub-ligase field for cancer treatment (31).Target protein degradation technology is a new field in the discovery and development of new drugs (126). PROTAC is an effective degradation tool of endogenous protein developed in recent years, which can control the tumor growth. The first oral drugs of PROTAC drug, ARV-471 and ARV-110, have achieved a good result in clinical trials (127). In addition to being a potential drug, PROTAC can be combined with the advantages of gene silencing or editing techniques, and provides a good tool to study protein function (128).With the progress of target protein degradation technology, it is expected to make new contribution to the development of new drugs by complementation with small molecule inhibitors.

## 5 Conclusion and perspectives

Lung cancer is an important chronic disease of human beings, with the poor five-year survival rate and the heavy medical burden. It is very important for healthy about the ubiquitination-deubiquitination balance of endogenous proteins under the internal environment changes of the body. The abnormal changes of protein ubiquitination are significantly associated with multiple diseases such as lung cancer. E3s in Ub-proteasome system has strong specificity to the substrate protein, which determines the ubiquitination of the target protein. Proteasome, enzyme E1, enzyme E2, enzyme E3, and deubiquitinases are potential sites to develop targeted therapies for lung cancer. Ubiquitination of proteins is diverse. Compared to single chain ubiquitination, the modification of multiple short-chain ubiquitination residues is more conducive to protein degradation. For lung cancer, the up-regulated E3 gene were mainly involved in protein polyubiquitination and proteasome-mediated ubiquitin-dependent protein catabolic process, and the down-regulated E3 genes were mainly involved in protein polyubiquitination, negative regulation of defense response, and proteasome-mediated ubiquitin-dependent protein catabolic process. Ubiquitination of cancer protein or deubiquitination of tumor suppressor protein is beneficial to the inhibition of lung cancer. The ubiquitin system is a treasure trove of drugs for the treatment of lung cancer, especially for E3 and proteasome inhibitors. Following small molecule protein inhibitors, precision protein degradation technology provides a promising approach to develop new drugs for lung cancer.

## Author contributions

ZY collated and analyzed literatures, prepare tables and figures, and wrote manuscript draft. JY and HJ participated in literature analysis and preparation of figures. XZ conceived the concept, critically revised the manuscript, and were responsible for its corresponding works. All authors contributed to the article and approved the submitted version.
